# A molecular test based on RT-LAMP for rapid, sensitive and inexpensive colorimetric detection of SARS-CoV-2 in clinical samples

**DOI:** 10.1038/s41598-021-95799-6

**Published:** 2021-08-12

**Authors:** Catarina Amaral, Wilson Antunes, Elin Moe, Américo G. Duarte, Luís M. P. Lima, Cristiana Santos, Inês L. Gomes, Gonçalo S. Afonso, Ricardo Vieira, Helena Sofia S. Teles, Marisa S. Reis, Manuel A. Ramalho da Silva, Ana Margarida Henriques, Miguel Fevereiro, M. Rita Ventura, Mónica Serrano, Catarina Pimentel

**Affiliations:** 1 Instituto de Tecnologia Química e Biológica António Xavier, Universidade Nova de Lisboa, Av. República, 2780-157 Oeiras, Portugal; 2 Centro de Investigação da Academia Militar (CINAMIL), Unidade Militar Laboratorial de Defesa Biológica e Química (UMLDBQ), Av. Dr. Alfredo Bensaúde, 1849-012 Lisbon, Portugal; 3 Centro de Medicina Naval-Marinha Portuguesa, Alfeite, 2810-001 Almada, Portugal; 4 Hospital das Forças Armadas, Azinhaga dos Ulmeiros, 1649-020 Lisbon, Portugal; 5 Instituto Nacional de Investigação Agrária e Veterinária, I.P., Laboratório de Virologia, Av. República, Quinta do Marquês, 2780-157 Oeiras, Portugal

**Keywords:** Molecular biology, Infectious-disease diagnostics, SARS-CoV-2

## Abstract

Until there is an effective implementation of COVID-19 vaccination program, a robust testing strategy, along with prevention measures, will continue to be the most viable way to control disease spread. Such a strategy should rely on disparate diagnostic tests to prevent a slowdown in testing due to lack of materials and reagents imposed by supply chain problems, which happened at the beginning of the pandemic. In this study, we have established a single-tube test based on RT-LAMP that enables the visual detection of less than 100 viral genome copies of SARS-CoV-2 within 30 min. We benchmarked the assay against the gold standard test for COVID-19 diagnosis, RT-PCR, using 177 nasopharyngeal RNA samples. For viral loads above 100 copies, the RT-LAMP assay had a sensitivity of 100% and a specificity of 96.1%. Additionally, we set up a RNA extraction-free RT-LAMP test capable of detecting SARS-CoV-2 directly from saliva samples, albeit with lower sensitivity. The saliva was self-collected and the collection tube remained closed until inactivation, thereby ensuring the protection of the testing personnel. As expected, RNA extraction from saliva samples increased the sensitivity of the test. To lower the costs associated with RNA extraction, we performed this step using an alternative protocol that uses plasmid DNA extraction columns. We also produced the enzymes needed for the assay and established an in-house-made RT-LAMP test independent of specific distribution channels. Finally, we developed a new colorimetric method that allowed the detection of LAMP products by the visualization of an evident color shift, regardless of the reaction pH.

## Introduction

A robust population-scale testing strategy for SARS-CoV-2 based on rapid, reliable, decentralized and affordable diagnostic tests is of utmost priority to guide public health interventions. This testing approach aligned with measures such as mask wearing, frequent hand washing and social distancing may be enough to prevent and contain major outbreaks while COVID-19 vaccination programs are in progress.

The gold standard of COVID-19 testing is RT-PCR, which detects the genetic material of SARS-CoV-2 in nasopharyngeal (NP) samples. Although very reliable, RT-PCR diagnostics are complex, laborious and expensive, and its worldwide use caused, in the early stages of the pandemic, a shortage of reagents needed for sample collection and viral RNA extraction.

Loop-mediated isothermal amplification (LAMP) is a DNA amplification method that allows rapid and sensitive detection of a specific gene^1–3^. LAMP merged with reverse transcription (RT-LAMP) has been successfully used for the detection of several respiratory RNA viruses^4–8^, including SARS-CoV-2 (reviewed in^9^). RT-LAMP is a powerful alternative to RT-PCR due to its high specificity and sensitivity, cost-effectiveness, and fast turnaround time (typically 30 min). In RT-LAMP, the amplification of the genetic material of the virus occurs at a constant temperature and, therefore, diagnostic tests based on RT-LAMP can be carried out anywhere with basic resources, as they only require a heat block or a water bath set to a single temperature. The reaction products can be analyzed by means of conventional DNA-intercalating dyes, agarose gel electrophoresis, UV-light illumination, or real-time fluorescence^10^. Alternatively, end-point colorimetric readouts are also possible through the detection of reaction by-products, such as pyrophosphate and protons, which are released during DNA polymerization, after the incorporation of deoxynucleotide triphosphates. LAMP colorimetric methods detect the turbidity, triggered by the accumulation of magnesium pyrophosphate^1^, or color changes, occurring when complexometric indicators^3,11^, pH sensitive dyes^12^ or even DNA-intercalating dyes^13–15^ are incorporated into the reaction. The simple technical and instrumental requirements of colorimetric RT-LAMP tests make them extremely attractive for point-of-care (POC) use and implementation in low-resource settings. Colorimetric RT-LAMP has been successfully used for detection of SARS-CoV-2 in NP fluids from COVID-19 patients^15–24^.

Recently, it was shown that SARS-CoV-2 could be detected in the saliva of infected individuals, highlighting salivary tests as valuable alternatives for COVID-19 diagnosis^25–27^. Saliva-based testing has numerous advantages over NP sampling, especially in a mass screening scenario. It can be performed easily and non-invasively, thus minimizing patient discomfort, and it does not require specialized personnel or the use of protective equipment, which saves time and reduces costs. For these reasons, saliva RT-PCR and RT-LAMP tests for SARS-CoV-2 detection have been widely explored in recent months^28–31^.

In the current study, we have established and evaluated a RT-LAMP colorimetric test for SARS-CoV-2 detection from RNA samples extracted from the NP fluid, or directly from the saliva, of COVID-19 patients. We have also developed a new colorimetric detection method based on a complexometric indicator that, when merged to LAMP, is capable of detecting SARS-CoV2 with great analytical sensitivity. In addition, we have produced the enzymes needed for the test and implemented an in-house-made assay fully independent of commercial reagents.

With this work, we join efforts with many other authors who, in the last months, have been testing and validating alternative tests for the detection of SARS-CoV-2 in order to make the molecular diagnosis of COVID-19 more accessible and to facilitate its large-scale implementation, even in settings that lack economic or infrastructural resources.

## Results

### Sensitivity of two different RT-LAMP colorimetric setups

The main components of the RT-LAMP colorimetric reaction are two enzymes (a reverse transcriptase (RT) and a strand displacement polymerase), a colorimetric dye (phenol red) and a primer set (typically composed of six primers)^12^. To detect SARS-CoV-2 using RT-LAMP, we took advantage of the primer set previously validated in vitro by Zhang et al*.*^24^ and tested, on clinical specimens from large cohorts of COVID-19 patients, by several other authors^16–19^. The primer set (N-A) targeted the N gene, which encodes the nucleocapsid protein and has the most abundant expression of subgenomic mRNA during infection^32–35^.

**Figure 1:**
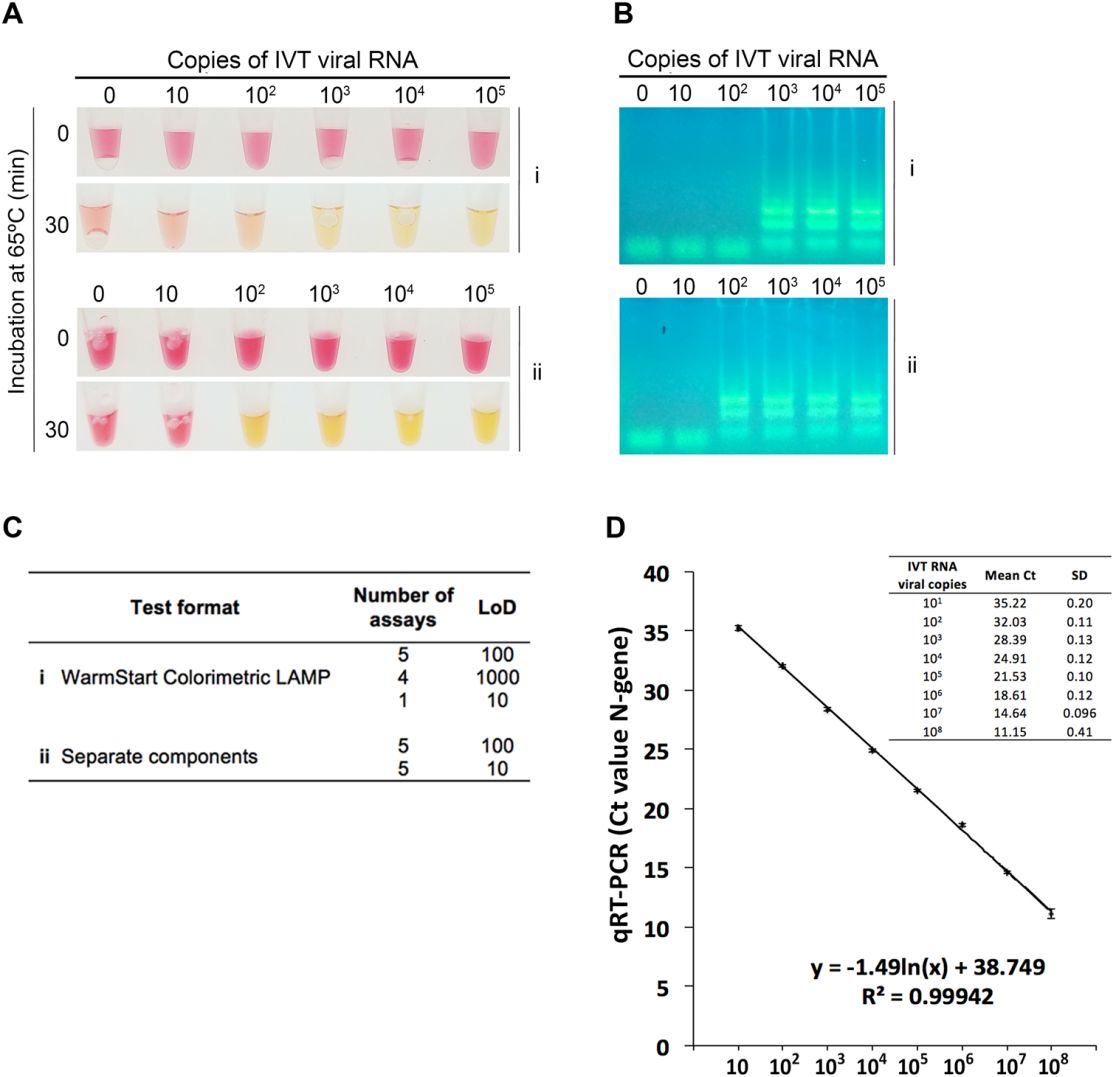
Limit of detection of the two different RT-LAMP formats and of RT-PCR. (**A**) A known number of copies of in vitro transcribed (IVT) viral RNA (N-gene) were amplified and detected by colorimetric RT-LAMP using the (i) WarmStart Colorimetric LAMP 2 × Master Mix (New England Biolabs) or (ii) the separate components (enzymes purchased individually and an in-house-made colorimetric buffer). The reactions were incubated at 65 °C for 30 min. (**B**) 10 μL of the RT-LAMP reaction were resolved in an agarose gel (2%) electrophoresis. The ladder pattern corresponds to the expected LAMP amplification pattern. (**C**) Limit of detection of ten replicates of the two test formats. (**D**) Standard curve generated by plotting the number of IVT RNA copies (x-axis) vs. the mean of the corresponding RT-PCR threshold cycle (Ct) value (y-axis) of three independent experiments (Original gel images in Fig. S1).

We tested two different assay formats. In one format, we used the WarmStart Colorimetric LAMP 2 × Master Mix (New England Biolabs), which includes all the reagent components with the exception of the primers. In the other, we purchased the separate enzymes (RTx and *Bst* 2.0) from New England Biolabs, while the reaction buffer with the colorimetric dye (phenol red) were prepared in-house as described by Tanner et al.^12^. The analytical sensitivities of these two setups were evaluated and compared by assaying in parallel tenfold serial dilutions of an in vitro transcribed N-gene RNA standard (IVT RNA), starting from 10^5^ copies down to 10 copies (per 20 μL reaction), at tenfold intervals (Fig. 1A). Color changes from pink (negative) to yellow (positive) were registered after a 30-min incubation period at 65 °C, as we found that for extended periods (up to 60 min), negative controls often turned yellowish. The amplification of the IVT RNA was confirmed by agarose gel electrophoresis (Fig. 1B). Ten replicates were analyzed per assay format (Fig. 1C) and IVT RNA dilutions were simultaneously analyzed by RT-PCR (Fig. 1D). The limit of detection (LoD) was reliably found to be between 100–1000 viral copies for the assay using the WarmStart Colorimetric LAMP 2 × Master Mix (Fig. 1A), whereas for that using the separate components the LoD was consistently one Log10 lower (10–100 copies). However, for half of the replicates, a tenfold lower LoD was achieved for both test formats (Fig. 1C). Such stochastic detection efficiency has been reported by others (1), and therefore we defined 100–1000 (WarmStart Colorimetric LAMP 2 × Master Mix) and 10–100 (reaction with separate components) as the robust limits of detection.

For the same serial dilution range, the RT-PCR assay was able to consistently detect down to 10 copies per reaction (mean Ct = 35.22) (Fig. 1D). Compared to RT-PCR, the RT-LAMP assay, depending on the test setup, detected up to ten- or one 100-fold less copies of viral RNA. As the RT-LAMP format using the separate components was consistently more sensitive, we decided to choose this setup in subsequent assays.

### Sensitivity and specificity of the colorimetric RT-LAMP assay in detecting viral RNA from the nasopharyngeal fluid

**Figure 2:**
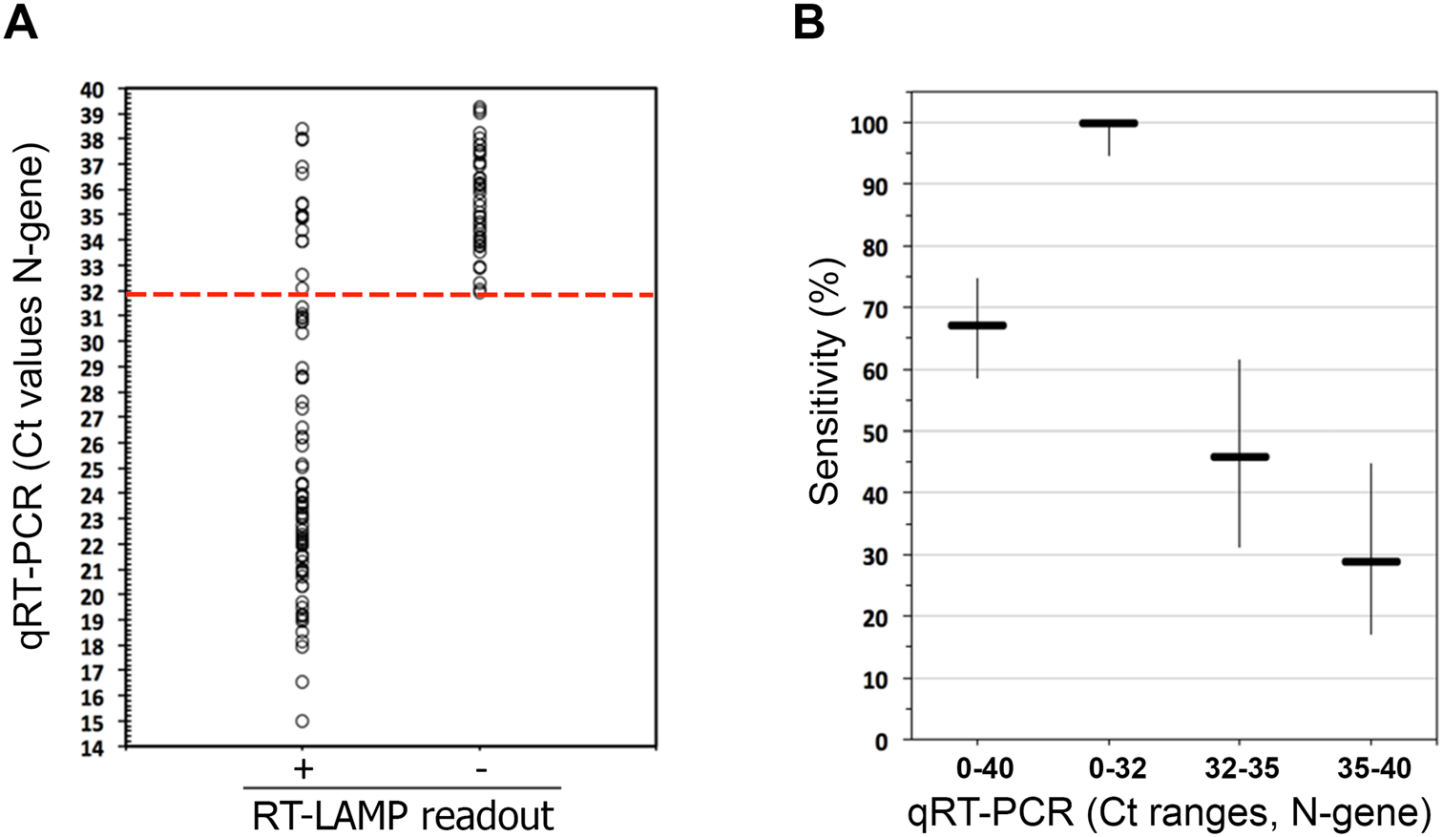
Detection of SARS-CoV-2 in NP samples using RT-LAMP. (**A**) Comparison of RT-LAMP and RT-PCR results. The Ct values (RT-PCR results) of 126 COVID-19 positive patients (y-axis) were compared to the RT-LAMP readout (x-axis) taken after 30 min of incubation at 65 °C (positive, +/yellow; negative, −/pink). The dotted red line indicates the Ct below, which there is 100% agreement between RT-LAMP and RT-PCR. (**B**) Sensitivity of the RT-LAMP assay across different ranges of Ct values (which reflect different viral loads). The thicker horizontal lines indicate the specificity calculated for the indicated Ct range (according to the data of panel (**A**) and Table 1). The vertical lines indicate the corresponding 95% confidence intervals.

We investigated whether the RT-LAMP assay, using separate components, could be used to accurately detect SARS-CoV-2 in clinical samples. For that purpose, we tested a set of surplus RNA samples extracted from the nasopharyngeal (NP) fluid of 177 individuals who were previously tested for COVID-19, using the standard clinical RT-PCR testing. The samples comprised 126 RNA samples that tested positive (RT-PCR positive, Ct ≤ 40) and 51 samples that tested negative (RT-PCR negative, Ct ≥ 40). As shown in Fig. 2A, after incubation for 30 min at 65 °C, a pink to yellow color change was visualized in all RT-LAMP reactions estimated to have more than 100 RNA molecules present in the reaction (RT-PCR positive, Ct ≤ 32, Fig. 1C), which is in agreement with the observed experimental sensitivity (Fig. 1A). We found two false positives, i.e. two RT-PCR negative samples that scored positive in the RT-LAMP assay (Table 1). Thus, the overall specificity of the assay was 96.1% (CI 87–99%) and the sensitivity for samples with Ct ≤ 32 was 100% (CI 94.7–100%). For lower viral load, as measured by RT-PCR (Ct > 32), the assay showed a decrease in diagnostic sensitivity (Table 1, Fig. 2B).

**Table 1:** RT-LAMP sensitivity across different ranges of Ct values.

RT-PCR (Ct value range)**	RT-LAMP		
	True positives	False negatives	% Sensitivity (95% CI)*
[0–40]	84	42	67.2 (58.56–74.81)
[0–32]	68	0	100 (94.65–100)
[32–35]	17	20	45.95 (31.04–61.62)
[35–40]	11	27	28.95 (17–44.76)

*Wilson’s binominal confidence interval.

**Ct values rounded to the nearest unit.

Overall, these results indicate a robust performance of the colorimetric RT-LAMP assay across a broad range of purified RNA samples.

### Sensitivity of the colorimetric RT-LAMP assay in detecting SARS-CoV-2 in saliva samples

We next optimized our RT-LAMP assay for direct detection of SARS-CoV-2 in saliva samples. To reduce the risk associated with handling samples containing infectious viral particles, saliva was self-collected into a tube and placed at 95 °C for 30 min, for inactivation. This simple heat inactivation procedure has been shown to enable an effective genetic detection of SARS-CoV-2 by other authors^30,31^. After a brief centrifugation step that significantly improved assay reliability (data not shown), the supernatant was diluted with TE, to buffer basal pH differences in saliva, and immediately analyzed or stored at − 80 °C. Lalli et al*.* have shown that TE is LAMP-compatible and does not affect the assay sensitivity^29^.

**Figure 3:**
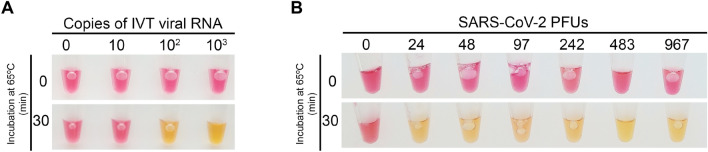
Limit of detection of the saliva RT-LAMP assay. Spike-in experiments of a healthy donor saliva with (**A**) tenfold dilutions of in vitro transcribed (IVT) viral RNA (N-gene) and (**B**) in vitro propagated SARS-CoV-2 virions. Saliva samples were processed as described in “Materials and methods” and 2 μL were analyzed by colorimetric RT-LAMP. *PFUs* plaque forming units. Figures are representative of three independent experiments.

We determined the LoD of the assay using both the IVT RNA standard and viral SARS-CoV-2 particles spiked into healthy human saliva to simulate clinical samples. We were able to detect 100 IVT RNA copies (Fig. 3A) and 24 SARS-CoV-2 viral particles (Fig. 3B) per reaction in only 30 min after inactivation, using our RT-LAMP protocol. Since at this sensitivity the assay would detect the typical viral load of SARS-CoV-2 found in the saliva of COVID-19 patients (100–1000 genomes per μL)^36^, we proceeded to test the clinical samples.

**Figure 4:**
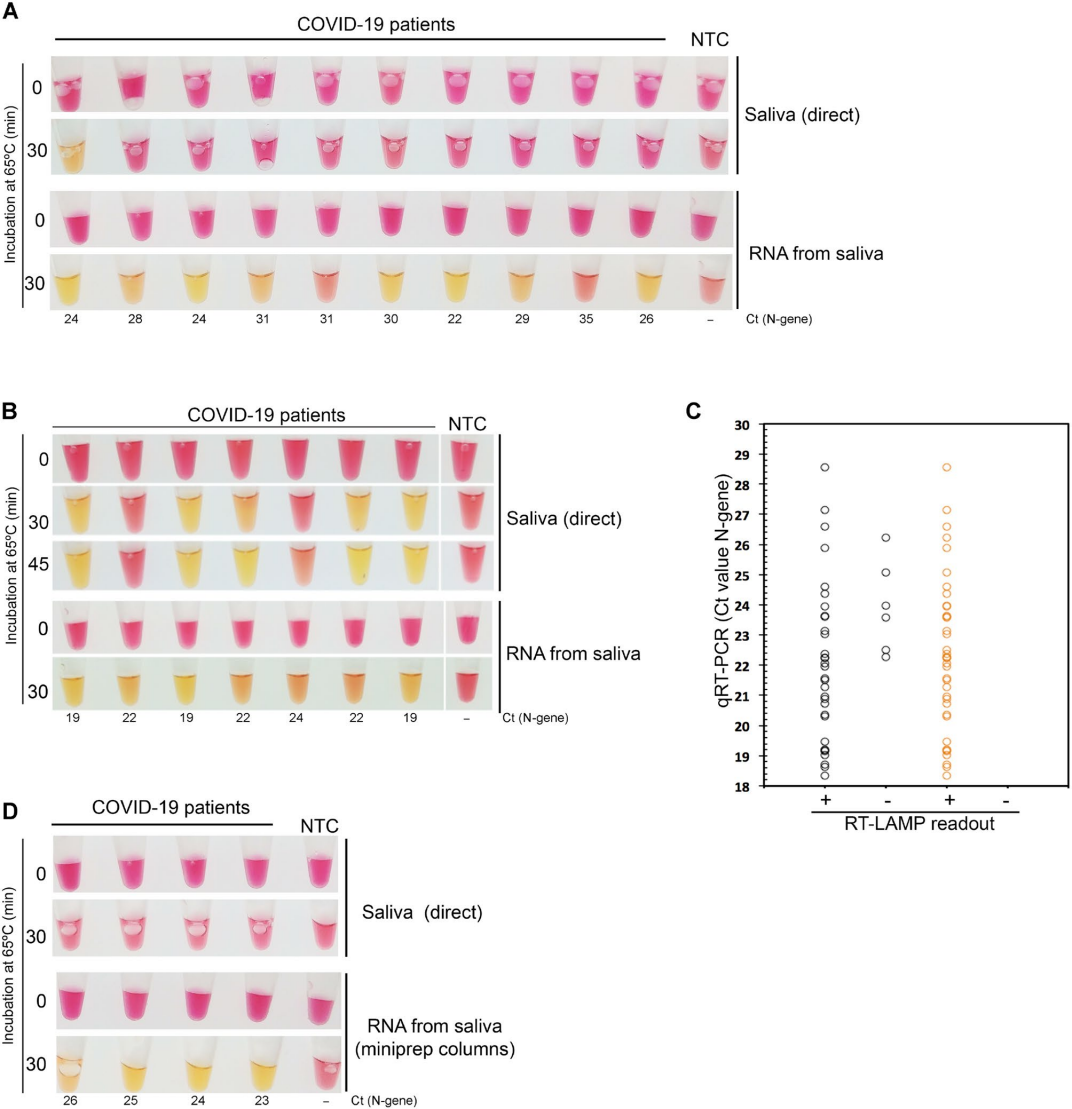
Detection of SARS-CoV-2 in saliva samples using RT-LAMP. RT-LAMP analysis of saliva samples of confirmed COVID-19 patients who (**A**) induced or not (**B**) salivation before sample collection. (**C**) Comparison of RT-LAMP and RT-PCR results. The Ct values (RT-PCR results) of 39 COVID-19 positive patients (y-axis) were compared to the RT-LAMP readout of the matched saliva samples (x-axis), after 30 min of incubation at 65 °C (positive, +/yellow; negative, −/pink). Black circles—direct saliva; orange circles—RNA extracted from saliva. (**D**) RNAs from the saliva of false negative samples (as determined by the direct saliva test) were extracted using plasmid DNA miniprep columns (ZR Plasmid Miniprep-Classic Kit, Zymo Research) and re-analyzed. *NTC* no template control. Cts (N gene) obtained for the paired NP samples are indicated.

Saliva and matched NP swab specimens of 49 individuals infected with SARS-CoV-2 (as previously determined by RT-PCR) were collected and analyzed by RT-LAMP (saliva) and RT-PCR (NP fluid). In addition, 15 saliva samples of healthy donors were tested by RT-LAMP. Saliva samples were self-collected as described above, and individuals were asked not to eat or drink before testing. A set of 10 of the 49 COVID-19-positive patients was asked to induce salivation by placing the tongue on the salivary sublingual glands. For this group, we could only detect SARS-CoV-2 sequences in the saliva of one patient using the direct RT-LAMP assay (Fig. 4A). However, after RNA extraction, 8 out of 10 individuals were identified as being SARS-CoV-2-positive. Taking into account the Ct values obtained for the paired NP samples (Fig. 4A), we put forward the hypothesis that by stimulating salivation we were diluting the saliva viral load, which might have accounted for a high number of false negatives. Corroborating this idea, for all other positive samples where salivation was not induced, we obtained a good correlation with the RT-PCR results (Fig. 4B,C), as 33 out of 39 samples were identified as positive samples, with no false positives registered. Therefore, the direct RT-LAMP assay had a sensitivity of 85% (CI 70–93%) for saliva samples with matched NP swabs with Ct ≤ 28 (Fig. 4C). Reaction volumes, but not saliva amounts, were scaled up to increase the assay sensitivity (Fig. 4B).

All saliva samples that were falsely negative by direct RT-LAMP were positive after RNA extraction (Fig. 4B). This step increases by 4–9 times the estimated cost of the assay (1€). Inspired by the work of Yaffe et al*.*^37^, to keep RT-LAMP affordable, we tested whether we could use silica columns routinely used in molecular biology laboratories to purify bacterial plasmids (mipreps), to extract viral RNA from saliva samples. As shown in Fig. 4D, false negative samples were found to be positive after RNA purification using this method, with an estimated cost per RT-LAMP test of 2€.

### Development of an in-house-made colorimetric RT-LAMP

Aiming to establish a colorimetric RT-LAMP test fully independent of commercial suppliers, we produced the two enzymes needed for the assay and benchmarked them against commercial alternatives using IVT RNA of SARS-CoV-2.

**Figure 5:**
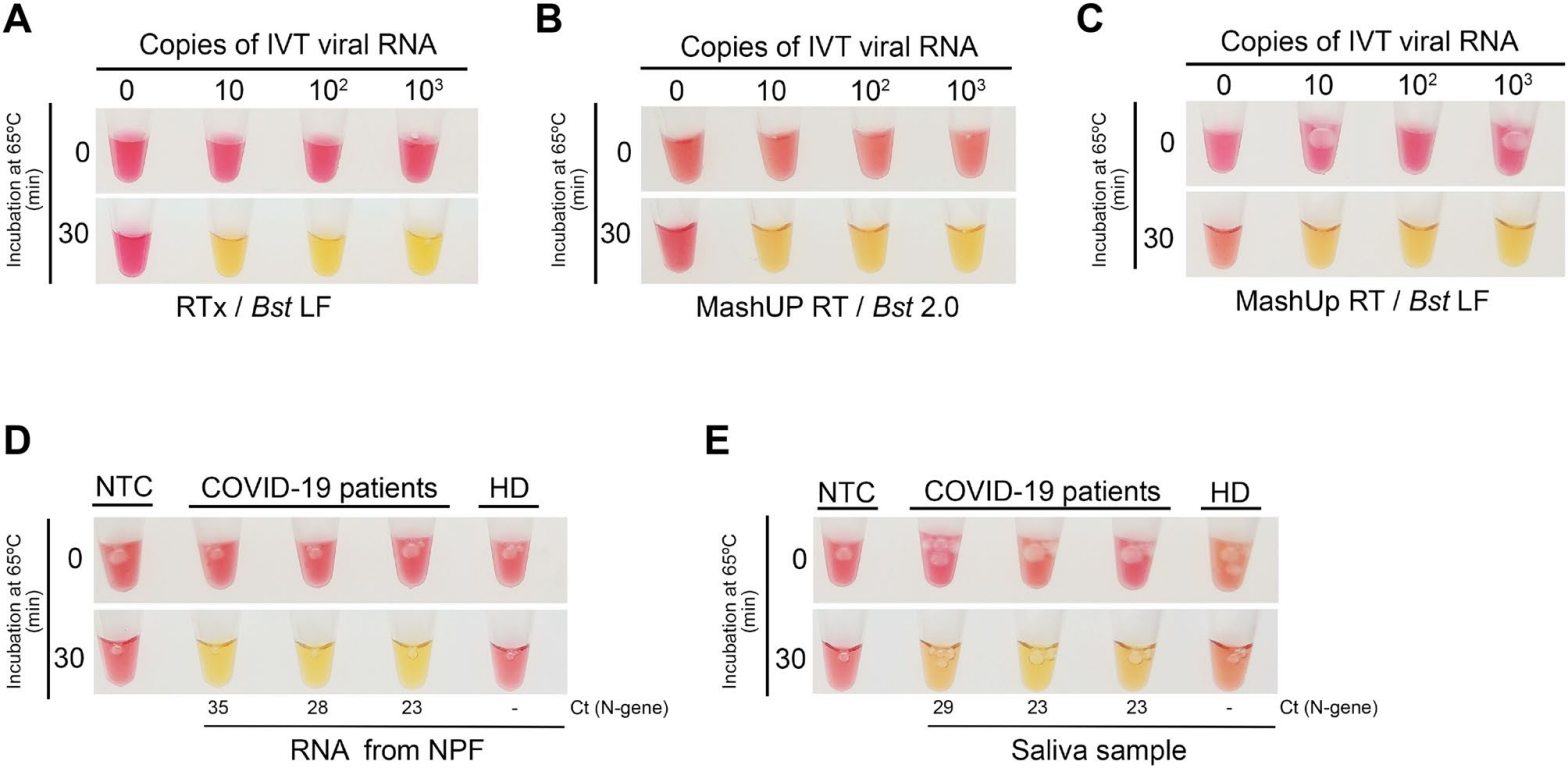
Analytical sensitivity of an in-house-made colorimetric RT-LAMP assay. Tenfold dilutions of in vitro transcribed (IVT) viral RNA (N-gene) were amplified via RT-LAMP and detected using a colorimetric buffer together with (**A**) RTx (New England Biolabs) and *Bst *LF (homemade), (**B**) MashUP RT (homemade) and *Bst *2.0 (New England Biolabs) or (**C**) MashUP RT (homemade) and *Bst *LF (homemade). The in-house-made setup was next used to detect SARS-CoV-2 sequences in (**D**) RNAs extracted from the NP fluid (NPF) and (**E**) saliva samples of COVID-19 positive patients. The reactions were incubated at 65 °C for 30 min. In (**D**) and (**E**) the Cts (N gene) obtained for the paired NP samples are indicated. *NTC* no template control, *HD* healthy donor. Figures are representative of three independent experiments.

As for the strand displacement polymerase, the gene encoding the large (Klenow) fragment of *Geobacillus stearothermophilus* was synthesized, with codon optimized for expression in E*. coli*, and inserted into the pET28 + vector. After a simple 2-step purification protocol, we ended up with 250 μL of *Bst* LF, at a concentration of 7.6 mg/mL. We next determined the LoD of the assay combining 1 μL of the purified *Bst* LF, 50-fold diluted (0.15 μg per 20 μL reaction), the in-house-made colorimetric reaction buffer, and RTx (New England Biolabs). This semi-commercial assay consistently detected 1–10 copies of the SARS-CoV-2 N gene per reaction (Fig. 5A). We found that, under our colorimetric conditions, *Bst* LF outperformed *Bst* 2.0 (New England Biolabs) (Fig. 1A and 5A). The amount of the produced *Bst* LF was enough to perform 12,500 tests at that analytical sensitivity (1–10 copies).

Alternatives to the commercial RTx were also explored. We started by testing several non-thermostable reverse transcriptases (from NZYtech and Roche), but it was not possible to detect LAMP products with an acceptable sensitivity (less than 10^6^ viral IVT RNA copies per reaction, data not shown). We also expressed and purified the MashUP RT enzyme (clone available at https://pipettejockey.com) that, when combined with *Bst* 2.0, was able to detect down to 10 IVT viral RNA copies (Fig. 5B), a LoD similar to the one obtained with the commercial enzyme (Fig. 5A). The MashUP purification consists of a single-step protocol, and sufficient enzyme was obtained to perform 500 assays (0.5 μL corresponding to 3.4 μg/μL were used directly in the reaction).

Finally, we combined the produced enzymes (*Bst* LF and MashUP) with the homemade colorimetric reaction mixture and assessed (i) the LoD of the assay (Fig. 5C) and, as proof of concept, (ii) whether this setup could identify SARS-CoV-2 N-gene sequences in the RNA extracted from the NP fluid and saliva of COVID-19 patients (Fig. 5D,E). Our in-house-made assay successfully detected SARS-CoV-2 viral sequences in all the three COVID-19 patients’ samples (Fig. 5D). Moreover, when using patients’ saliva, processed as described above, instead of NP RNA, the assay was also capable of identifying SARS-CoV-2 infected patients (Fig. 5E). Corroborating the work of Alekseenko et al*.*^38^, these results clearly indicate that, using simple expression and purification protocols and home-made buffers, it is possible to establish a colorimetric assay, fully independent of specific supply chains, that efficiently detects SARS-CoV-2 RNA sequences from clinical specimens.

### A new colorimetric method for detection of RT-LAMP amplification products

**Figure 6:**
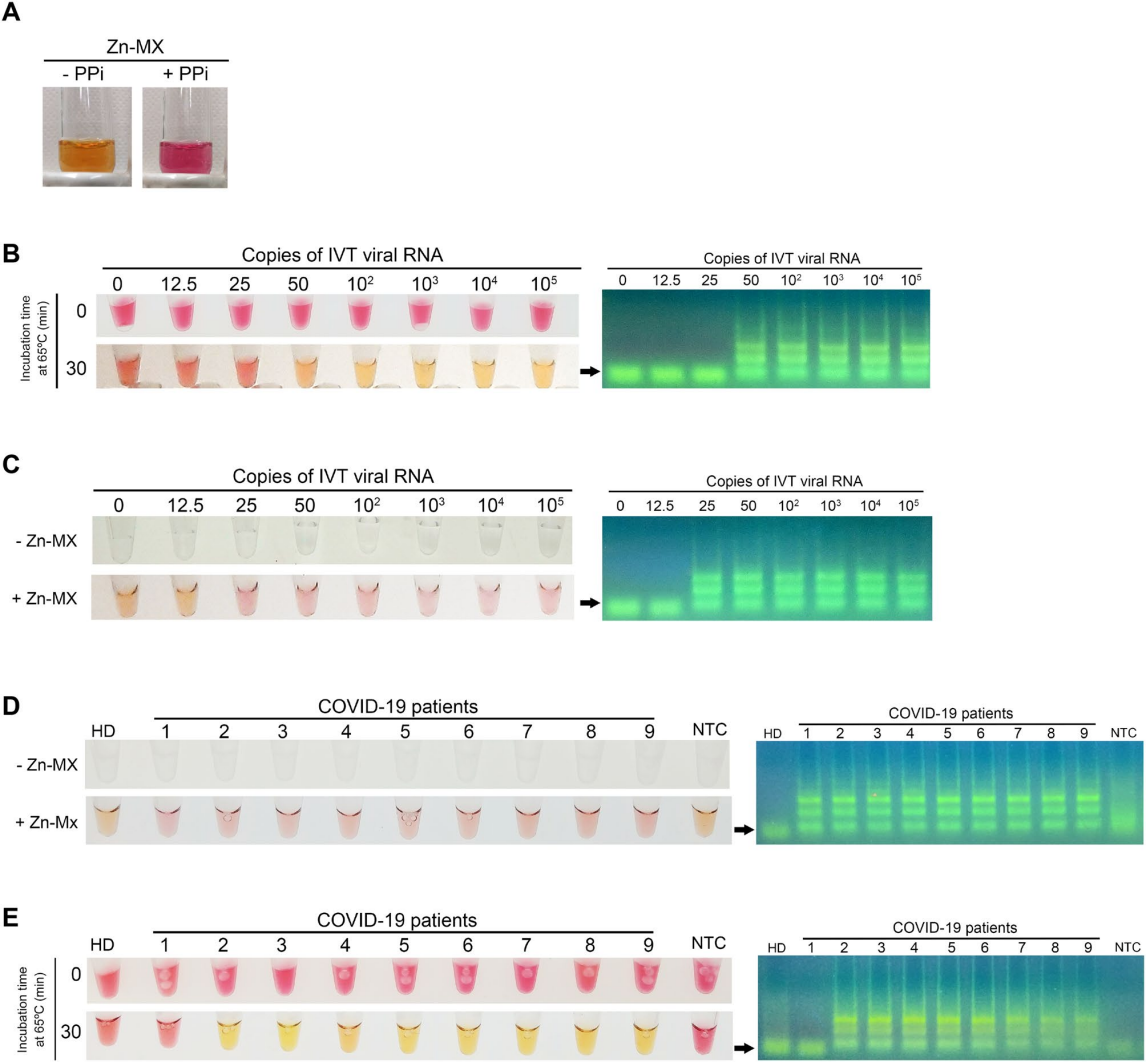
Alternative colorimetric detection based on the complex murexide-zinc. (**A**) A strong color change from yellow to pink is observed when pyrophosphate (PPi) is added to a solution containing Zn-MX. Tenfold dilutions of in vitro transcribed (IVT) viral IVT RNA (Ngene) were amplified via RT-LAMP and detected using phenol red (**B**) or Zn-MX (**C**). Amplification was confirmed by agarose gel electrophoresis (AGE). Saliva samples of a healthy donor (HD) and of nine COVID-19 patients were analyzed by RT-LAMP followed by detection with Zn-MX (**D**) or phenol red (**E**) and amplification was confirmed by AGE (Original gel images in Fig. S1).

The strong and evident color shift observed with phenol red renders this pH-sensitive dye much preferred for end-point colorimetric detection of LAMP products. However, when the phenol red method is used with crude samples, interference of the sample pH with the assay readout is often observed. Indeed, when establishing the direct RT-LAMP saliva protocol, we had to discard one sample due to the initial acidification of the reaction, as a strong color shift to yellow was observed immediately after sample addition into the reaction mixture. Although several colorimetric indicators are available for detection of LAMP products^3,11–15^, the pale color shift they produce, which is difficult to distinguish by the naked eye, has certainly restrained their wide use. To overcome these limitations, we developed a new colorimetric detection method based on the complexometric indicator, murexide (MX), which forms a complex with divalent zinc (Zn^2+^)^39^. In the absence of Zn^2+^, MX has a pink color, whereas in the presence of the divalent cation it turns yellow. Because pyrophosphate (PPi) forms a strong complex with zinc, we reasoned that the PPi released during DNA polymerization would displace Zn^2+^ cations from MX, inducing a color change from yellow to pink. By mimicking the reaction components in a test tube containing the Zn-MX complex, an evident color shift from yellow to pink was observed immediately after PPi addition (Fig. 6A). Unfortunately, we found that Zn, but not MX, strongly inhibited the LAMP reaction (data not shown), making it impossible to use Zn-MX in a one-step colorimetric assay. Therefore, after an incubation period at 65 °C for 30 min, the tubes were opened and MX (0.5 mM) and ZnCl_2_ (2.5 mM) were added to the reaction. To avoid carryover problems due to the post-amplification opening of the tubes, this step was performed in a separate room.

Using the in-house produced enzymes, we first compared the sensitivity of Zn-MX with that of phenol red using IVT RNA (Fig. 6B,C). Like phenol red, Zn-MX showed an evident color difference depending on the presence (pink) or absence (yellow) of LAMP amplification. Moreover, the method enabled the clear detection of SARS-CoV-2 in crude saliva samples of nine COVID-19 positive patients (Fig. 6D), whereas with phenol red the viral genetic material was only identified in eight of these samples (Fig. 6E).

## Discussion

Widespread testing, preferably based on different supply chains, is required to curtail the ongoing pandemic. To address that need, we have in this work evaluated a LAMP-based colorimetric test to rapidly detect SARS-CoV-2 in RNAs extracted from patient’s NP fluids, using a single tube protocol. The assay also allows for detection of the virus directly from patient´s saliva with minimal processing and increased protection of the testing personnel. We also showed that using simple expression and purification protocols together with homemade buffers, it is possible to establish an inexpensive colorimetric assay, fully independent of specific supply chains, that efficiently detects SARS-CoV-2 RNA.

While not as sensitive as the reference diagnostic method for COVID-19, RT-PCR, the simplicity, turnaround time and low associated costs of our test make it an attractive and efficient tool for infection control. According to existing literature, the LoD of the test is sufficient to identify individuals with viral titers high enough to transmit the virus (300–1000 viral copies per μL)^27,40,41^. This test sensitivity is understood to be adequate for surveillance and screening of the asymptomatic population. The availability of such a testing solution is therefore of great importance, as infectiousness peaks occur before or at the symptoms onset^42^. Indeed, the rapid evolution of COVID-19 has been partly attributed to transmissions occurring through people who are presymptomatic or asymptomatic^43^; efforts to implement a strategy enabling communities to test asymptomatic individuals require urgent attention and testing tools to support it.

Several authors have recently shown that the use of different primer sets boosts RT-LAMP sensitivity, possibly due to better primer efficiency and/or higher target abundance. Also different saliva treatment protocols, combining certain chemicals and proteinase K, have been shown to improve SARS-CoV-2 detection in saliva samples^16,21,22,28,29,31^. Thus, we reason that there is still room to improve the sensitivity of our test.

As expected, RNA extraction greatly improved the saliva test sensitivity, by increasing the concentration of the viral sequences in the sample. Many other authors have reported similar findings^21,22,44,45^ and extensive efforts have been made to establish alternative protocols that enable RNA enrichment using fast and inexpensive methodologies^21,22^. Here we showed that RNA extraction using common plasmid DNA extraction columns is an economical way to concentrate and purify viral RNA from saliva samples.

To eliminate the impact of acidic saliva samples on the test readout, we have developed a new colorimetric reading, independent of changes in the pH of the LAMP reaction. The method uses a divalent zinc salt (such as ZnCl_2_) and the complexometric indicator murexide to form a transient complex (Zn-MX). The presence of PPi, a by-product of the reaction, is indicated by the indicator displacement method, since Zn^2+^ forms a more stable complex with PPi and thus releases murexide. As the presence of zinc inhibits the amplification reaction, the metal can only be added at the end of the reaction, thus requiring the tubes to be opened post-amplification. This procedure poses the threat of carryover contaminations, very common in LAMP reactions^46,47^, which leads to false positives. We therefore do not anticipate that the Zn-MX method, in its current formulation, can be used routinely in a molecular diagnostic laboratory. However, the molecular saliva-based tests currently available for COVID-19, whose workflow already demands opening the LAMP reaction tube, may certainly benefit from our method^48^. Additionally, the method can be safely used in closed systems using microfluidic diagnostic cartridges, similar to the one recently described by Ganguli et al.^49^.

Overall, this study, while addressing some of the testing bottlenecks imposed by the current pandemic, reinforces RT-LAMP as a powerful method for sensitive and inexpensive molecular diagnosis of COVID-19 that can be easily deployable in limited resource settings.

## Materials and methods

### Sample collection, processing and storage

Clinical specimens were collected at Hospital das Forças Armadas and processed in Laboratório de Bromatologia e Defesa Biológica (Unidade Militar Laboratorial de Defesa Biológica e Química). Saliva specimens (~ 1 mL) were self-collected into sterile tubes (50 mL or 1.5 mL). Patients were asked not to eat or drink before testing. NP swab-matched samples were collected in parallel and placed in 3 mL Universal Viral Transport Media. Tubes containing clinical specimens were decontaminated with an alcohol-based solution and identified. After collection, samples were kept at 4 °C for 2–4 days or processed immediately. Samples were inactivated by incubation at 95 °C for 5 min (NP swabs) or 30 min (saliva samples). Salivas were centrifuged at 5000*g* for 5 min and 200 μL of the supernatant were diluted in TE 10 × (1 ×, final concentration) and frozen at − 80 °C until analysis. The saliva pellets were also frozen.

### RNA extraction from clinical samples

Total viral RNA was extracted from 140 μL of NP deactivated samples using Viral RNA Mini Kit (QIAGEN) and eluted in 60 μL of RNAse free water, to ensure the RNA elution buffer has no impact of pH in RT‐LAMP reactions. As for saliva samples, total RNA (from the pellets) was isolated using the RNeasy Mini Kit (QIAGEN) following the manufacturer’s instructions or the LogSpin method^37^ as described by the authors. Briefly, the pellet was mixed by vortexing with 250 μL a guanidine-based solution (8 M guanidine-HCl, 20 mM MES hydrate and 20 mM EDTA). The mixture was centrifuged at 16,000*g* for 5 min and the supernatant was mixed with 250 μL of 100% ethanol, and loaded into the ZR plasmid miniprep columns (ZYMO Research). The column was washed twice with 450 μL of 3 M Na-Acetate and 320 μL of 70% ethanol. RNA was eluted in 30 μL of water.

### SARS-CoV-2 RNA standard

To prepare the SARS-CoV-2 RNA standard, the N gene was amplified from the plasmid 2019-nCoV_N_Positive Control (Integrated DNA Technologies) with a T7-promoter-containing primer (5′-TAATACGACTCACTATAGGatgtctgataatggaccccaaaa-3′) and the reverse primer (5′-ttaggcctgagttgagtcagc-3′), then the product was in vitro transcribed using the HiScribe T7 High Yield RNA Synthesis Kit, NEB), according to the manufacturer’s instructions. Template DNA was removed using Turbo DNase (Invitrogen) and RNA was then purified using the RNeasy Mini Kit (QIAGEN). Standard RNA copy numbers were calculated from concentration measured using Take3 from Epoch from Biotek and confirmed using a Ultrospec2100pro (Amersham Biosciences).

### Virus isolation and spike experiments

SARS-Cov-2 isolate, BetaCoV/Portugal/ICV1006/2020, was obtained at INIAV from a patient confirmed positive for SARS-CoV-2 by RT-PCR. Virus isolation and production of the virus stock were accomplished in Vero E6 cells (African green monkey kidney cells, catalog no.ATCC CRL-1586) maintained in Eagle’s minimum essential medium (MEM) supplemented with 10% fetal bovine serum (FBS), penicillin (100 U/mL) and streptomycin (100 mg/mL), at 37 °C in a 5% carbon dioxide atmosphere. The infectivity titer of the viral stock prepared from infected cell culture supernatants was determined by a standard plaque assay. Aliquots of saliva (500 μL) were spiked with decreasing numbers of plaque forming units (pfus) of isolate ICV1006 and used to evaluate the limit of detection of the saliva RT-LAMP assay.

### RT-PCR

SARS-CoV-2 N-gene and an internal control (RNase P) were amplified by RT-PCR using the TaqMan 2019-nCoV Assay Kit v1 (Termofisher) with TaqMan Fast Virus 1-step Master Mix (Termofisher) and the CFX96 thermocyler (BioRad), according to the manufacturer’s instructions.

### RT-LAMP assays

RT-LAMP reaction was performed in a total volume of 20 μL containing the following components: 8 U *Bst* 2.0 (NEB), 7.5 U RTx (NEB) and 1 × colorimetric buffer mix [1.6 μM FIP/BIP primers, 0.4 μM LF/LB primers, 0.2 μM F3/B3 primers Gene N-A^24^, 10 mM (NH_4_)_2_SO_4_ (Merck), 50 mM KCl (BDH), 8 mM MgSO_4_ (BDH), 0.1% Tween 20, 0.2 mM Phenol Red (Sigma), 1.4 mM each dNTP (NZYTech)]. For the in-house-made assay, we used the same colorimetric buffer mix, 0.5 μL of MashUP RT (6.8 mg/mL) and 1 μL of *Bst* LF (7.6 mg/mL) 50 × diluted. WarmStart colorimetric LAMP 2 × master mix (M1800S, NEB) was also used with the above final primer concentration.

When the complexometric indicator MX-Zn was used, samples were assembled as described above, but without phenol red. After 30 min, 2 μL of 5 mM Murexide and 1 μL of 50 mM of ZnCl_2_ were added to the reaction, in a post-LAMP workspace. All reactions were performed in a thermocycler at 65 °C and pictures were taken at the indicated time points. Figures depicting the readout of the RT-LAMP assays are representative of three independent experiments.

### Zinc-murexide colorimetric method

All reagents obtained from commercial sources in analytical grade. Analytical solutions were prepared in ultrapure grade water from a Milli-Q system, as follows: MOPS buffer pH = 7.4 at 20 mM, magnesium chloride (MgCl_2_) at 47.5 mM, zinc chloride (ZnCl_2_) at 47.1 mM, sodium pyrophosphate (Na_4_P_2_O_7_) at 50 mM, ATP at 25 mM, and murexide (MX) at 0.5 mM. The MX solution was prepared immediately before use or otherwise kept frozen. Samples (1 mL), simulating the starting conditions of the RT-LAMP assay, contained 8 mM of magnesium chloride and 1.4 mM of ATP, buffered at pH = 7.4 with 10 mM of MOPS. To these samples were added a few drops of a MX solution to attain a suitable color intensity, which turned the samples violet, indicating that MX was in the free form. Addition of ZnCl_2_ at 8 mM to the samples rendered them orange, indicating a change of the indicator to its complexed form. Finally, titration of pyrophosphate into the samples caused a color change back to pink from ca. 16 mM, pointing to a release of the indicator caused by the binding of zinc to pyrophosphate. These color changes demonstrated that MX is a suitable colorimetric indicator to detect pyrophosphate in presence of magnesium (Supplementary Figure S1).

### Expression and purification of Bst1 klenow

The gene encoding the klenow fragment of *Bst*1 (UniProt sequence P52026, residue 291–876) was synthesized (codon optimized for expression in *E. coli*) and inserted into the pET28 + vector with nucleotides encoding an N-terminal 6HisTag and a TEV cleavage site (Genescript). The resulting plasmid was used for transformation of *E. coli* BL21 (DE3) pLysS. Overnight pre-cultures (10 mL) were grown at 37 °C and used to inoculate 1 L Power Broth (Molecular Dimensions) with 100 µg mL^−1^ ampicillin and 50 µg mL^−1^ kanamycin. The culture was grown at 37 °C until OD_600_ reached 0.7–0.9. At this point, the culture was moved to 18 °C and expression was induced by adding 0.5 mM isopropyl-β-d-thiogalactopyranoside (IPTG). After overnight expression, the cells were harvested by centrifugation at 7548*g* for 30 min at 4 °C, flash frozen and stored at − 20 °C. Upon protein purification, the cells were resuspended in 20 mL extraction buffer [150 mM NaCl, 50 mM Tris–HCl pH 7.5, 10 mM MgCl_2_, 1 mg/mL DNase I, 1 mg/mL lysozyme and one tablet EDTA free proteinase inhibitor (Roche)] and subjected to multiple freeze/thaw cycles (alternating room temperature water bath and liquid nitrogen). The lysate was cleared by centrifugation at 48,385*g* for 30 min at 4 °C and the supernatant was carefully removed and added to a 5 mL HisTrap HP purification column (Cytiva), previously equilibrated in buffer A (150 mM NaCl, 50 mM Tris–HCl pH 7.5). The protein was eluted over a 10 CV gradient from 5 to 100% buffer B (buffer A with 0.5 M Imidazol). Fractions containing Bst1 Klenow were identified by SDSPAGE, pooled and dialyzed overnight in 2 L buffer A in the presence of TEV (1:20) at 4 °C. The dialyzed and TEV cleaved protein was thereafter added to a 5 mL HisTrap column and eluted in the Flow Through (due to the removal of the HisTag). The HisTag free Bst1 Klenow was thereafter desalted through a HiTrap desalting column (Cytiva) followed by a final purification step on a 5 mL HiTrap Heparin HP column (Cytiva), to remove eventual residual DNA bound to the protein. The protein was eluted over a 10 CV gradient in buffer B2 (buffer A and 1 M NaCl). Fractions containing BstKlenow was identified by SDSPAGE, pooled, concentrated to 7.6 mg/mL by Amicon Ultra-15 concentration filter units (10 kDa cut off, Millipore) and stored at − 80 °C.

### Expression and purification of MashUP reverse transcriptase

The MashUp RT plasmid (kindly provided by https://pipettejockey.com), which encodes a modified Feline Leukemia Virus Reverse Transcriptase (RT) and plasmid pGTf2 that encodes for a chaperon were co-transformed into *E. coli* BL21 (DE3) competent cells and plated on L-Broth (LB) agar (NZYTech) plates containing 50 μg/mL kanamycin and 30 μg/mL chlorophenicol. Overnight cultures were inoculated with fresh transformants and grown at 37 °C, 150 RPM in LB selective medium. Subsequently, the overnight culture was diluted 100 × in Terrific Broth (TB). The cells were grown at 37 °C, 150–170 RPM until OD 600 nm reach 0.8–1.0. Then, temperature was lowered to 18 °C and protein expression induced with 0.5 mM IPTG and 5 ng/mL tetracycline, for the RT and chaperone, respectively, and grown additionally for 18 h at 18 °C. The cells were harvested by centrifugation at 4500×*g* for 10 min at 4 °C and resuspended in MashUp-RT lysis buffer (25 mM Tris–HCl pH 8, 300 mM NaCl, 10% glycerol, 40 mM imidazole, 0.5% Triton X-100), supplemented with one tablet of Complete EDTA-free protease inhibitor cocktail (one unit per 1 L). Cells were disrupted by French press and the extract was clarified by centrifugation at 100,000×*g*, 90 min at 4 °C. The supernatant was loaded into an IMAC column equilibrated with lysis buffer. The column was washed with the same buffer and the adsorbed proteins were eluted from the column with 25 mM Tris–HCl pH 8, 300 mM NaCl, 10% glycerol, 500 mM imidazole, 0.5% Triton X-100. Protein was concentrated in an Ammicon ultrafiltration device with a 30 kDa cutoff. Total protein present in the sample was quantified by BCA assay (6.8 mg/mL) using albumin as a standard.

### Ethics statement

The Director of the Hospital das Forças Armadas (HFA) approved all experimental procedures, which were carried out following the guidelines of the HFA Ethics Committee. The study was conducted in accordance with the European Statements for Good Clinical Practice and the declaration of Helsinki of the World Health Medical Association. Informed consent was obtained from all participants.

## Supplementary Information

Supplementary Figure S1.
