# Supplementary material for: A molecular test based on RT-LAMP for rapid, sensitive and inexpensive colorimetric detection of SARS-CoV-2 in clinical samples
Source: Sci Rep. 2021 Aug 12;11:16430. doi: 10.1038/s41598-021-95799-6 (PMC8361189; doi:10.1038/s41598-021-95799-6)
Supplement: Supplementary file 1 — Supplementary Figure S1. [file 41598_2021_95799_MOESM1_ESM.pdf]

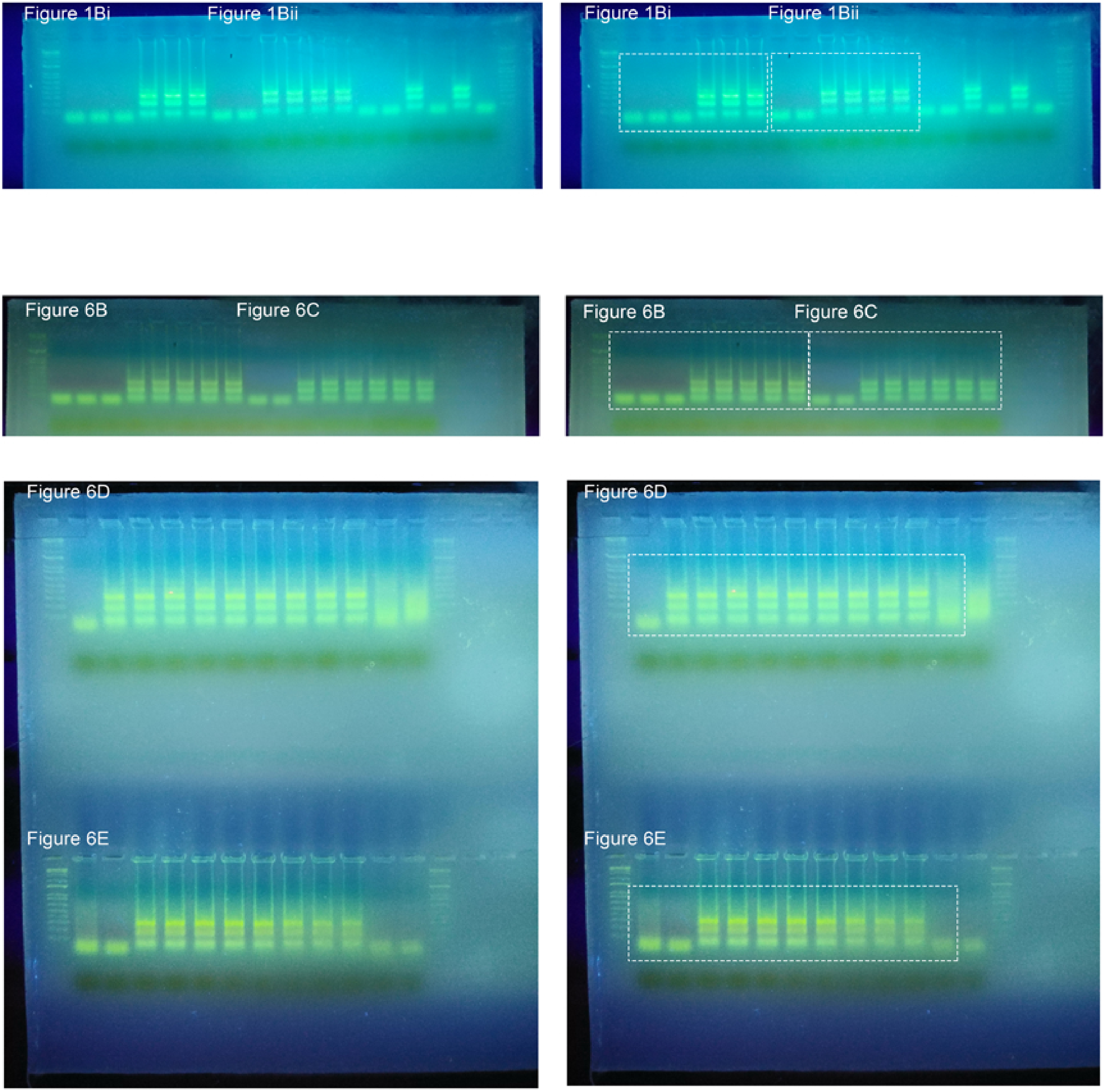

Figure S1- Full length gels of Figures 1 and 6. 10 µL of the RT-LAMP reaction were resolved in an agarose gel (2%).electrophoresis stained with GreenSafe (NZYTech). Dashed lines indicate the cropped aereas of the gels.
